# A Treatise on the Principles and Practice of Medicine

**Published:** 1887-03

**Authors:** 


**A Treatise on the Principles and Practice of Medicine.** Designed for the use of Practitioners and Students of Medicine. By AUSTIN FLINT. M. D., LL.D., late Professor of Principles and Practice of Medicine and Clinical Medicine, Bellevue Hospital College, New York. Sixth edition. Revised and largely re-written by the author, assisted by Wm. H. Welch, M. D., Professor of Pathology, Johns Hopkins University, Baltimore. Philadelphia: Lea Brothers & Co. 1886.

There is, perhaps, no work on the principles and practice of medicine which has had as great an influence on the practice of English-speaking physicians as "Flint's Practice." Certainly no American physician was better qualified to be a teacher than its distinguished author, who, in the year of his death, completed his fiftieth regular course of lectures on this subject. This last edition was re-written and revised by his own hand, and will be looked upon as the crowning work of a glorious and successful life. In addition to Dr. Flint's revision, his able colaborators have added many new chapters, among which deserving of special mention are the articles upon syphilitic disease of the lungs and cerebral syphilis, general pathology of fevers, and a full consideration of recent discoveries concerning the bacterial origin of various infectious diseases. The work now admirably represents the existing state of the science of medicine, and no important discovery in pathology nor clinical medicine has failed to receive due recognition.

